# Home-Isolation Care in Newly COVID-19-Positive Elderly Patients: A Caregiver-Centric Explanatory Framework

**DOI:** 10.3389/ijph.2023.1606060

**Published:** 2023-07-19

**Authors:** Arista Lahiri, Sweety Suman Jha, Arup Chakraborty, Abhijit Dey, Madhumita Dobe

**Affiliations:** ^1^ Dr. B. C. Roy Multi-Speciality Medical Research Centre, Indian Institute of Technology Kharagpur, Kharagpur, West Bengal, India; ^2^ Department of Community Medicine, Medical College and Hospital, Kolkata, West Bengal, India; ^3^ WHO-NTEP Technical Support Network, Swasthya Bhawan, Kolkata, West Bengal, India; ^4^ Foundation for Actions and Innovations Towards Health Promotion (FAITH), Kolkata, West Bengal, India; ^5^ All India Institute of Hygiene and Public Health, Kolkata, West Bengal, India

**Keywords:** mixed-methods, elders, elderly, home isolation, health behavior

## Abstract

**Objectives:** This community-based study aimed to identify the effect of different behavioral factors of family caregivers on the decision for home-isolation-based treatment of a new COVID-19-diagnosed elderly individual. It also explored the facilitators and barriers contributing to the decision-making process.

**Methods:** A mixed-methods design was adopted to study the role of behavioral constructs such as risk tolerance, risk aversion, regret aversion, loss aversion, self-efficacy, and risk perception in healthcare-seeking decisions. By integrating the findings from the quantitative and qualitative parts, a framework was developed.

**Results:** Self-efficacy, risk perception, and risk tolerance related to different issues were crucial factors behind the healthcare decision. However, regarding the various issues under consideration, risk perception followed by risk tolerance were the significant predictors for decision-making.

**Conclusion:** To enhance appropriateness and equity in emergency healthcare-seeking, interventions should target risk tolerance and risk perception, taking into account the awareness levels of caregivers and the target population’s risk and regret aversion. Such integrated approaches can improve the quality of care for elderly patients in home-based settings.

## Introduction

Home-based care of the elderly by family caregivers is an efficient method of cost-effective, personalized, and compassionate care in terms of personal care, preventive and early diagnostic services, health education, basic therapeutic measures, and transitional home care [1, 2]. In India, the population is aging, and as per projection, by 2030, the number of elderly (≥60 years) will rise to 198 million [3]. Considering the socio-economic variation, healthcare access needs and preferences, and substantial treatment/healthcare costs, geriatric healthcare is challenging for the health system [4]. Through capacity-building of the family members, home-based care provision for the elderly becomes crucial, as these unpaid caregivers often shoulder the responsibility of primary decision-making for better care of the elderly [5]. However, home-based care should also integrate the need for institutional care when required, especially in the Indian context and other resource-constrained settings.

### Context of the Study

The issue of elderly healthcare came into further prominence with the start of the COVID-19 pandemic because of its high elderly mortality [6–9]. During the early phases of the pandemic, amidst the resource constraints in low-and middle-income countries (LMICs), community beliefs, discrimination, stigma, and patient and family caregiver preferences further complicated the prioritization of available treatment resources and differential access to healthcare. In the state of West Bengal, several health advisories for COVID-19 management were issued for comprehensive healthcare for the elderly. The initial guidelines suggested institutional care for aged individuals diagnosed with COVID-19 illness often irrespective of the severity. In contrast, the authorities subsequently advocated institutional care for the elderly with symptoms or, in some cases, moderate disease [10–13].

The current study was conducted in several areas of West Bengal during the peak of the first and second pandemic waves in India when the caseloads were also high in the state (Supplementary Figure S1). General awareness about the disease, including its warning symptoms, individual preference, trust in the healthcare system, the perceived ability to manage a situation (i.e., self-efficacy), risk perception and acceptance, and normative influences of the home-based family caregiver play an important role in healthcare-seeking behavior, particularly in the pandemic context [14–20]. However, due to the fatal nature of this disease, especially in the case of elderly patients, the loss and regret aversion biases of caregivers also affect health-related decisions, especially treatment decisions [21, 22]. Thus, from a behavioral perspective, the multi-factorial nature influencing healthcare-seeking and associated choice-making becomes interesting to understand.

### Purpose of the Study

The discussion of healthcare-seeking decisions is critical to understanding the infrastructural requirements and resource needs of vulnerable groups during emergencies for an optimum yield and avoiding health catastrophe. Still, there is a lack of evidence regarding the different factors influencing the choice of treatment modality for COVID-19, i.e., home isolation or institutional treatment in the case of older people. The most widely used healthcare service utilization and decision-making model is the Andersen Model, which conceptually focuses on the interplay of different behavioral issues and environmental factors leading to a particular healthcare service utilization by the beneficiaries [23]. The model has also been expanded to effectively understand the elderly population’s uptake of different healthcare services [24]. While these models are pertinent in understanding standard healthcare-seeking decision-making processes, their applicability in emergencies or crises remains inconsistent conceptually. To address this gap, this study examined the effect of different behavioral factors of the caregivers that led to the decision for home-isolation-based treatment of newly diagnosed COVID-19-positive elderly individuals. The facilitators and barriers contributing to such treatment decisions were further qualitatively explored to shape the role of different behavioral constructs into an explainable healthcare seeking model that can be utilized in emergencies and crises like the COVID-19 pandemic.

## Methods

### Design and Setting

A community-based mixed-methods study was conducted in three selected sub-urban Municipal areas surrounding Kolkata: Barrackpore, Madhyamgram, and Serampore, during the first and second waves of the COVID-19 pandemic in India. In these three areas, 9, 9, and 10 municipal wards were selected, respectively, and these were considered clusters for analysis (Supplementary Figure S2). The data for this study were collected between September and October 2020 (the first wave peaked during September 2020) and April and June 2021 (the second wave peaked during May 2021, although daily new case numbers documented a steep rise from April 2021). In this convergent parallel mixed-methods study, the quantitative part was a case-control study with 1:1 allocation, and the qualitative part comprised exploratory interviews with the participants.

### Participants

The study was conducted among home-based family caregivers of newly COVID-19-positive elderly individuals during the study period that resided permanently in the study area (i.e., at least for 10 years or more). Caregivers aged ≥18 years were included, while those with active COVID-19 illness during data collection were excluded. In the case of repeat infection in the elderly, the caregiver was also excluded. Where there were multiple home-based caregivers for an elderly individual, the primary caregiver (i.e., the person making most of the decisions related to healthcare) was selected for the study. For the quantitative part, i.e., the case-control study, newly COVID-19-positive elderly who remained in home isolation for at least 24 h following the diagnosis were considered the ‘cases’ in home isolation. Newly COVID-19-positive elderly individuals admitted for institutional care (in any government or private healthcare institution) within the first 24 h following diagnosis were considered the “control” group in this study. The *households* in each cluster were the sampling unit in this study, and one newly diagnosed COVID-19-positive elderly patient and their primary caregiver were selected from each household. For the quantitative part, based on the findings of a prior pilot survey, the sample size per group, considering a case:control ratio of 1:1, power of 99%, a 95% confidence level, and 10% incomplete/partial response, was 1,472 [25]. Finally, complete response data from 1,392 controls (hospital admission) and 1,412 cases (home isolation management) were included in the study. For the qualitative part, the participants from the case and control groups were purposively selected. Participants who agreed to participate in the study and completed the quantitative survey were considered for the qualitative interviews.

### Quantitative Measurement

#### Study Tool

For the quantitative part of the study, the respondents (home-based caregivers) were interviewed with a pre-designed pre-tested structured questionnaire. The questionnaire consisted of questions regarding the socio-demographic details of the elderly patient and also of the respondent (caregiver); the caregiver’s awareness of the requirements of home-isolation treatment; the clinical profile of the patient; the caregiver’s self-efficacy, risk perception, risk tolerance, and normative influencers of home-isolation therapy for the elderly.

In the first phase of questionnaire development, the items about the behavioral determinants of treatment decisions were generated and pooled together. The pooled items were then assessed by a panel comprising experts from the fields of Public Health (two), Health Economics (two), and Psychology (one). A final version with relevant items was generated based on consensus among the experts by excluding redundant questions and incorporating items that were not included initially but were subsequently considered appropriate by the experts (content validity ratio: 0.82). The questionnaire was then pre-tested on a sample of 53 home-based caregivers of elderly patients (who earlier had COVID-19 infection). The vital quantitative constructs considered in this study for choosing home-isolation treatment were self-efficacy beliefs, risk perception, and risk tolerance of home-isolating the infected elderly.

The questionnaire was translated into Bengali and Hindi (the local languages) by language experts and was back-translated to English by separate experts. It was administered for the survey among the selected caregivers. The cases and controls were identified, and respondents (caregivers) were recruited consecutively based on the daily case list available with the community welfare organizations working in the study areas during the study period. During recruitment, frequency matching was done according to the gender and age group of the elderly patient with COVID-19. The field workers from the local welfare organizations conducted quantitative data collection at the time of their house visits. Informed written consent was obtained from the participants. For 76 controls and 198 cases, the interview was difficult during house visits, so telephonic interviews were conducted. The COVID-19 prevention protocols were followed during the house visits for data collection.

#### Self-Efficacy of Home-Isolation

Self-efficacy beliefs (SE) comprised of the caregiver’s confidence regarding the arrangement of a separate/isolated area for the patient at home, giving required medicines at home as and when needed, providing clinical monitoring at home, obtaining a clinical opinion from a doctor as and when required, arrangement of admission to a hospital any time if the condition of the patient gets worse, getting help from neighbors/friends/relatives as and when required, and bearing the expenses for home-isolation management of the patient. Each item was measured on a scale of 1 (not confident at all) to 5 (very much confident). The Cronbach’s alpha calculated for the SE scale was 0.82.

#### Risk Perception Associated With Home-Isolation

Risk perception (RP) was measured by the caregivers’ perception regarding the severity of the patient’s condition, the chance of deterioration in home isolation, treatment availability at home, the chance of incurring a serious cost for treatment if kept in home isolation, susceptibility of other family members, disease severity among family members (if infected), the chance of social discrimination, and the chance of difficulty in admission to hospital/nursing home later on. The items were measured on a scale of 1 (not at all) to 5 (very much). For the RP scale, Cronbach’s alpha was noted to be 0.78.

#### Risk Tolerance to Home-Isolation

The items for measurement of risk tolerance (RT) were the caregiver’s willingness to keep the patient in home isolation when having serious complications at home [e.g., Shortness of Breath (SOB)], the chance of developing serious complications over time, inadequacy of treatment at home, treating the patient at home incurring a serious cost, the patient spreading the disease to others, considering other family members if those infected develop severe illness, non-availability of hospital beds in future, and experiencing social discrimination. The RT items were measured on a scale of 1 (not willing at all) to 5 (very much willing). For the RT scale, Cronbach’s alpha was noted to be 0.87.

### Qualitative Measurements

The qualitative component included 35 in-depth interviews (IDIs) with the caregivers. Among these IDIs, 20 IDIs were with caregivers of home-isolated patients, and 15 were with home caregivers of already hospitalized patients. Two researchers (AL and SSJ) with prior experience and training in qualitative research methods conducted the IDIs. Each IDI lasted for 20–30 min in strict adherence to the interview guide developed beforehand. All the IDIs were conducted after obtaining informed written consent for participation. The IDI guide was prepared from the brainstorming sessions with three subject experts. Initially, a pool of questions was developed under different issues for qualitative interviews. Next, based on expert consensus, a few questions with associated probes were selected for the IDI guide. The guide was further validated by five experts from the disciplines of Public Health (two), Health Economics (two), and Psychology (one). Issues such as the caregiver’s perception regarding the disease, the caregiver’s feelings about the patient, perceived challenges and facilitators of home-based caregiving to the patient, and the caregiver’s views on the appropriate management strategy for the elderly COVID-19 patient were elicited through the IDIs. Data collection continued until data saturation when no new information was yielded from the interviews.

### Analysis

The quantitative responses were analyzed in STATA 14.2 (StataCorp, College Station, TX, United States), and the qualitative data were analyzed by the hand code technique. The responses to each Likert-type item in the SE, RP, and RT domains were scored from 1 to 5. The negative statements were reverse scored so that a higher score in SE, RP, and RT items indicated higher self-efficacy, risk perception, and risk tolerance, respectively. The mean (± standard deviation) scores were calculated for each item in the SE, RP, and RT domains separately for cases and controls and compared with the help of an independent sample *t*-test. The caregiver’s awareness about home-isolation management, normative influencers of treatment decisions, and the clinical profile of the elderly were multiple response items. They were reported in terms of proportions in cases and controls. These items were compared among the cases and controls with the help of the Chi-squared test, and Bonferroni’s correction was used to account for multiple comparisons.

A mixed-effects multi-level logistic regression model was developed to determine the effect of different predictors on the choice of home-isolation-based treatment for the elderly, with the municipality as the highest level, the clusters of participants nested within the municipalities, and the individual participants nested in the clusters. On *post hoc* analysis, the intra-cluster correlation at levels of the municipality and participant clusters were <0.001 and 0.207, respectively. The model was built using maximum likelihood estimation, and robust standard errors were used. Models were found to be statistically appropriate through an indicative conservative likelihood ratio (LR) test (P_χ_^2^ < 0.001) [26]. The model adjusted for the socio-demographic profile of the home-based caregivers to determine the effects of the caregiver’s awareness about home-isolation management, the patient’s clinical profile, normative influencers, risk perception and tolerance, and self-efficacy beliefs of home-isolation management. Effects were calculated using an adjusted odds ratio (aOR) with a 95% confidence interval (95% CI).

For the IDIs, transcript generation and translation from the local language to English were done within a day of the interview. Data collection and coding to find the critical segments were done simultaneously. Transcripts were read multiple times initially to have a general understanding of the content. Two coders applied the hand code technique independently, and themes were generated. The third coder cross-checked the codes of the two primary coders and also resolved any discrepancies. Inter-coder agreement was, thus, established. Thematic analysis was done, where codes were merged and summarized to form themes, and the themes prepared were compared between the cases and controls.

The researchers (particularly AL and SSJ), owing to their responsibility of serving in different settings during the pandemic, spent a prolonged time in the field to adequately understand the settings and the participants, which ensured an accurate account of the participants’ narratives to lend credibility. Considering the deep engagement of the researchers in healthcare delivery for the elderly affected by COVID-19, the findings of the qualitative inquiry and its integration with quantitative findings were presented to an expert group comprising members from different specializations, with lived experience of hospitalization and home-based care. This peer debriefing exercise not only rigorously examined the reflexivity and addressed potential biases in the qualitative findings but also generated an interpretation of the transcripts and the findings other than that of the research team. Finally, by integrating the quantitative and qualitative findings, a new framework was proposed.

### Ethics

Ethical clearance was obtained from the Institutional Ethics Committee, Medical College and Hospital, Kolkata (MC/KOL/IEC/NON-SPON/730/07/2020 dt.07/07/20). All of the research was performed in accordance with the Declaration of Helsinki guidelines for research involving human subjects. Participants were recruited in the study after obtaining informed written consent. No incentives were provided to the participants for participation.

## Results

### Background Information and Clinical Profile

The socio-demographic profiles of the home-isolated and hospitalized patients in the quantitative part are shown in Supplementary Table S1. Most of the caregivers were aged 30–49 years and female. The mean age of the patients was 67.08 (±4.32) and 66.94 (±4.35) years in the isolated and hospitalized groups, respectively. The caregiver’s *per capita* monthly family income and educational status were significantly different between the two study groups. The home-isolated patients mostly (39.94%) belonged to the lowest income quartile (≤ INR 5,000.00), while a majority (44.76%) of the hospitalized patients had income in the third quartile (INR 8,000.01–13,750.00). The caregivers of the home-isolated patients had a comparatively higher educational status than the caregivers of the elderly who were hospitalized. Among the participants, 15 caregivers of hospitalized elderly and 20 caregivers of home-isolated elderly were included in the qualitative part of the study. The basic demographic information of the caregivers who were included in the qualitative part of the study is provided in Supplementary Table S2. The majority of the respondents of the IDIs were aged between 30–39 years, and the patients were mostly the parents of the caregivers. Supplementary Table S3 summarizes the clinical profiles of the patients in the two study groups. Overall, there were no statistically significant differences between the two groups regarding reported symptoms and co-morbidities. Fever and body ache were the most common symptoms reported by the patients. Hypertension was the most common co-morbidity.

### Awareness Regarding Home-Isolation Management

Table 1 outlines the caregivers’ awareness of the features of the illness and their influencers. Most caregivers in either study group recognized shortness of breath or a lowered oxygen saturation and drowsiness as vital warning signs of disease severity. They also acknowledged the importance of regular medication practices and regular measurement of oxygen saturation.

**TABLE 1:** Awareness about warning signs/symptoms of severe illness and requirements for keeping a patient in home isolation (West Bengal, India. 2021).

	Home isolation patients (*n* = 1,412)	Hospital admission patients (*n* = 1,392)	*p*-value^a^
Warning signs/symptoms recognized by the caregivers^b^			
Shortness of breath and/or oxygen saturation <94%	1,212 (85.84)	1,188 (85.34)	0.711
High fever	921 (65.23)	867 (62.28)	0.105
Diarrhea	904 (64.02)	917 (65.88)	0.304
Patient getting drowsy	1,181 (83.64)	1,122 (80.60)	0.036*
Patient having co-morbidities like hypertension, diabetes, etc.	761 (53.90)	730 (52.44)	0.441
Awareness about requirements for home isolation-based treatment^b^			
A separate/isolated well-ventilated room and bathroom is needed for the patient	1,381 (97.80)	1,342 (96.41)	0.027*
Patient must be under a doctor’s advisement	812 (57.51)	815 (58.55)	0.576
Patient must be given medications irrespective of symptom severity and/or other treatment regularly	1,251 (89.16)	1,200 (86.21)	0.017*
Patient must be given a nutritious diet	770 (54.53)	792 (56.90)	0.208
The oxygen saturation must be measured regularly	1,169 (82.79)	1,131 (81.25)	0.288
Normative influencers^b^			
Doctor/health personnel	621 (43.98)	631 (45.33)	0.472
Family members	1,081 (76.56)	1,085 (77.95)	0.381
Friends/relatives	389 (27.55)	366 (26.29)	0.453
Opinions/news in social media	763 (54.04)	779 (55.96)	0.305
Patient	491 (34.77)	505 (36.28)	0.405

“n” represents the number of participants in each study group. The figures within parentheses represent the column percentage of each cell. *statistically significant at *p* < 0.05.

^a^ *p*-values calculated by chi-squared test.

^b^ Multiple responses.

### Behavioral Beliefs of Home-Isolation Management

The results of the bivariate analysis of the items related to self-efficacy belief, risk perception, and risk tolerance of the caregivers concerning treatment decisions are depicted in Table 2. While there was not much difference in self-efficacy beliefs related to home-isolation treatment, caregivers of the hospitalized elderly had an overall higher risk perception compared to the caregivers of the home-isolated patients. The mean score for risk tolerance for social discrimination following home isolation treatment had a statistically significant difference.

**TABLE 2:** Self-efficacy, risk perception, and risk tolerance of the caregivers (West Bengal, India. 2021).

	Home isolation patients (*n* = 1,412)	Hospital admission patients (*n* = 1,392)	*p*-value^a^
Self-efficacy beliefs^b^			
Separate/isolated arrangements can be made at home	3.30 (±0.98)	3.24 (±0.99)	0.153
The patient can be given required medicines at home as and when required	3.33 (±1.04)	3.29 (±1.04)	0.318
The patient can be provided with clinical monitoring at home (e.g., BP, SPO_2_)	3.28 (±0.88)	3.21 (±0.86)	0.037*
Clinical opinion from a doctor can be obtained as and when required	3.29 (±0.90)	3.24 (±0.91)	0.183
The patient can be admitted to a hospital at any time if the condition gets worse	3.19 (±0.88)	3.16 (±0.87)	0.285
I can get help from my neighbors/friends/relatives as and when required	2.90 (±0.79)	2.86 (±0.80)	0.125
I can bear the expenses for home-isolation management of the patient	3.51 (±0.83)	3.43 (±0.82)	0.020*
Risk perception^c^			
How severe do you think is patient’s condition at the moment?	2.46 (±1.20)	2.64 (±1.19)	0.000*
Do you think the patient’s condition can deteriorate in home isolation?	3.32 (±1.10)	3.32 (±1.07)	0.992
Do you think all treatment required will be available at home?	2.98 (±1.22)	3.01 (±1.19)	0.585
Do you think keeping the patient in home isolation can ultimately incur a severe cost for treatment?	2.37 (±1.26)	2.62 (±1.31)	0.000*
Do you think there is any chance that others in the family will contract the disease because of the patient?	2.52 (±1.30)	2.92 (±1.27)	0.000*
Do you think your family members can develop severe complications if they contract the disease?	2.42 (±0.70)	2.38 (±0.66)	0.192
Do you think people may discriminate against you if you keep the patient under home isolation?	2.42 (±1.23)	3.13 (±1.36)	0.000*
Admission to a hospital/nursing home may not be possible in future	1.73 (±0.76)	3.59 (±1.40)	0.000*
Risk tolerance^d^			
The patient is having severe complications at home (e.g., SOB)	2.47 (±1.05)	2.40 (±1.07)	0.125
The patient may develop severe complications over time (e.g., SOB, or even may die at home without giving much time for treatment)	2.32 (±0.86)	2.32 (±0.83)	0.994
All the necessary treatments may not be provided at home	2.55 (±0.97)	2.53 (±0.92)	0.587
Treating the patient at home can incur a considerable amount of cost	2.85 (±1.06)	2.87 (±1.04)	0.517
The patient can spread the disease to others if kept at home	2.84 (±0.67)	2.85 (±0.67)	0.715
Any of the other family members, if infected, may develop severe disease	2.26 (±0.67)	2.27 (±0.64)	0.856
There may not be available beds in the future to admit the patient when required	2.42 (±1.02)	2.41 (±1.01)	0.872
The local people may discriminate if you keep the patient at home	2.75 (±1.28)	2.11 (±0.94)	0.000*

“n” represents the number of participants in each study group. The figures within parentheses represent the standard deviation of item scores according to the study group. *statistically significant at *p* < 0.05.

^a^ *p*-values calculated by a two-sample two-tailed *t*-test.

^b^ The confidence regarding the issues is measured on a scale of 1 (not confident at all) to 5 (very much confident).

^c^ Perceptions are measured on a scale of 1 (not at all) to 5 (very much).

^d^ Willingness to keep the patient in home isolation under certain situations is measured on a scale of 1 (not willing at all) to 5 (very much willing).

### Multi-Level Model

Table 3 shows the results of the multi-level modeling analysis of the various factors associated with the decision to home isolate the elderly COVID-19-infected patients instead of admitting them to hospitals. There were protective odds against home isolation in joint families and families with higher income quartiles. Certain patient-reported symptoms, caregivers’ better awareness, confidence over monetary ability, and comparatively higher risk tolerance were statistically associated with home-isolation treatment. However, the caregivers who were confident of getting admission when in need, and with mostly a higher risk perception but having higher risk tolerance towards the chance of a future increase in disease severity had a protective odds ratio against home isolating the elderly patients.

**TABLE 3:** Multi-level model of factors associated with the decision of home isolation treatment of the elderly patients^a^ (West Bengal, India. 2021).

Factors	aOR (95% CI)	*p*-value	Factors	aOR (95% CI)	*p*-value
Age of the caregiver [Ref.: < 30 years]			Normative influencers		
30–39 years	1.35 (0.65–2.82)	0.419	Doctor/health personnel	1.06 (0.79–1.44)	0.691
40–49 years	1.26 (0.59–2.66)	0.548	Family members	0.80 (0.56–1.14)	0.209
50–59 years	4.85 (0.53–44.51)	0.162	Friends/relatives	1.21 (0.85–1.71)	0.286
≥ 60 years	0.51 (0.04–7.34)	0.624	Opinions/news on social media	1.03 (0.76–1.39)	0.864
Sex of the caregiver [Ref.: Male]			Patient	0.85 (0.62–1.17)	0.322
Female	0.83 (0.56–1.22)	0.336	Self-efficacy beliefs		
Religion [Ref.: Hinduism]			The isolated arrangement at home	0.94 (0.77–1.15)	0.538
Islam	0.94 (0.60–1.47)	0.789	Provision of medicines at home	0.97 (0.80–1.16)	0.712
Others	0.81 (0.31–2.14)	0.673	Clinical monitoring of the patient at home	1.08 (0.82–1.41)	0.590
Education [Ref.: Graduate or above]			Obtaining a clinical opinion from a doctor when required	1.01 (0.81–1.26)	0.930
Completed Higher Secondary	1.18 (0.81–1.72)	0.381	Admission to a hospital if the condition worsens	0.77 (0.60–0.99)	0.040*
Completed secondary or below	1.10 (0.75–1.62)	0.615	Help from neighbors/friends/relatives as and when required	0.91 (0.68–1.21)	0.504
Type of family [Ref.: Nuclear]			Bearing the expenses for home-isolation	1.37 (1.09–1.73)	0.007*
Joint	0.67 (0.49–0.93)	0.017*	Risk perception		
Per capita monthly family income (in Rupees) [Ref.: ≤ 5,000.00]			Current severity	0.78 (0.66–0.92)	0.003*
5,000.01–8,000.00	0.25 (0.16–0.39)	0.000*	Possibility of deterioration	0.12 (0.09–0.16)	0.000*
8,000.01–13,750.00	0.04 (0.03–0.07)	0.000*	Treatment availability	1.02 (0.89–1.17)	0.739
>13,750.00	0.30 (0.19–0.48)	0.000*	Serious cost of treatment at home	0.63 (0.54–0.74)	0.000*
Currently an earning member			Other family members getting affected	0.81 (0.69–0.94)	0.005*
Patient	1.30 (0.70–2.40)	0.406	Any other family member may get severe illness if infected	1.44 (0.99–2.09)	0.054
Caregiver	0.76 (0.54–1.07)	0.111	Possibility of discrimination	0.54 (0.45–0.64)	0.000*
Warning symptoms/signs recognized			Hospital admission may not be possible in future	0.11 (0.09–0.13)	0.000*
Patient complaining of shortness of breath or oxygen saturation <94%	0.84 (0.54–1.30)	0.434	Risk tolerance		
High fever	0.95 (0.69–1.30)	0.738	The patient has serious problems	1.65 (1.24–2.20)	0.001*
Diarrhea	0.77 (0.57–1.06)	0.110	The patient may develop serious problems over time	0.48 (0.29–0.81)	0.006*
Patient getting drowsy	1.14 (0.78–1.68)	0.502	All treatments may not be provided	1.00 (0.79–1.27)	0.985
Patient having co-morbidities	1.18 (0.87–1.59)	0.290	Treating at home can incur considerable expenses	1.04 (0.89–1.21)	0.641
Awareness of requirements of home isolation			The patient can spread the disease to others	1.16 (0.88–1.53)	0.278
Separate/isolated well-ventilated room and bathroom needed	1.61 (0.66–3.88)	0.293	Other family members may develop severe illness if infected	0.78 (0.53–1.16)	0.219
The patient must be under a doctor’s advisement	1.07 (0.79–1.45)	0.641	Beds may not be available in future	0.79 (0.56–1.12)	0.185
The patient must be regularly given basic medications irrespective of symptom severity	0.95 (0.60–1.49)	0.811	The local people may discriminate	2.74 (2.26–3.34)	0.000*
The patient must be given a nutritious diet	0.74 (0.55–1.00)	0.052			
Oxygen saturation must be measured regularly	1.49 (1.01–2.20)	0.046*			
Current clinical symptoms					
Loss of taste	1.04 (0.75–1.43)	0.821			
Loss of smell	1.01 (0.74–1.38)	0.966			
Fever	1.54 (1.12–2.11)	0.007*			
Body-ache	0.77 (0.54–1.10)	0.148			
Shortness of Breath	0.89 (0.58–1.35)	0.581			
Diarrhea	0.84 (0.59–1.19)	0.326			
Co-morbidity present [Ref.: No diagnosed co-morbidity]	1.23 (0.76–1.99)	0.394			

*statistically significant at *p* < 0.05.

^a^ The multi-level model is developed, taking population clusters (i.e., municipal wards) nested within the municipalities included in this study. *p*-value for likelihood ratio test over logistic model: 0.000. Akaike’s information criterion = 1,383.264, Bayesian information criterion = 1751.47. Residual intraclass correlation at the municipality level is < 0.000 and 0.207 at the cluster level. aOR, adjusted odds ratio; 95% CI, 95% confidence interval of odds ratio, Ref., Reference category.

### Qualitative Findings

#### The General Perception of the Disease

**FIGURE 1:**
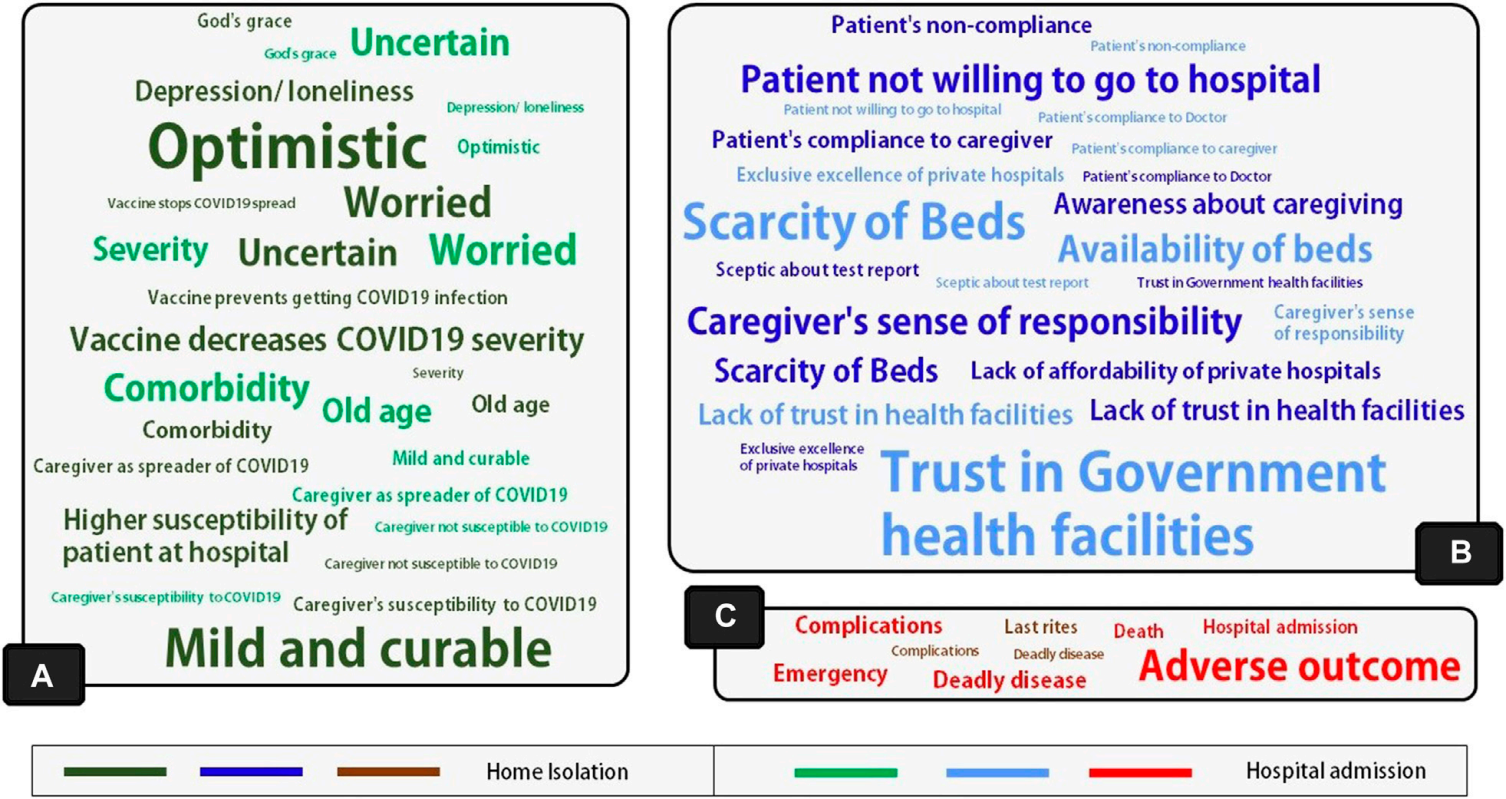
Word clouds showing key findings regarding **(A)** General perception about the disease and its outcome perceived susceptibility of the disease, and perception about the role of COVID-19 vaccines; **(B)** Caregiver’s perceived barriers and facilitators, and perception about the health system; **(C)** Risk and regret aversions related to the care of the patient (West Bengal, India. 2021). The font size of the words indicates the relative frequency of the themes separately for home-isolated and hospitalized individuals*.*

The general perception of the COVID-19 illness, its severity, and its susceptibility are depicted in Figure 1A. Caregivers of the elderly in home isolation were optimistic about the cure and mostly considered COVID-19 disease to be mild and curable. According to a 29-year-old male caregiver, “I have seen many people get cured without any treatment. Some of (my) father’s friends had COVID-19, and they got cured without anything….” They perceived that the vaccine decreases disease severity: “She had (COVID-19) vaccine 2 weeks back. She will overcome this. It’s not a big deal,” – confirmed a middle-aged male respondent while taking care of her mother in home isolation. However, another caregiver, a 44-year-old man, expressed his anguish over his father getting COVID-19: “I wonder how after taking the (COVID-19) vaccine, he is positive! He has no complaint…”

However, caregivers of hospitalized elderly patients were uncertain about the disease outcome and were, thus, worried. According to a 30-year-old man, who decided to hospitalize his 63-year-old father diagnosed with COVID-19, “…I am worried because of the symptoms. The oxygen crisis and all the news reports are causing all the more stress to me. Let’s see… If God saves him!” The caregivers were concerned about their patient’s age, co-morbidities, and severity.

#### Perception About Barriers and Facilitators of Patient Care

Figure 1B represents the barriers and facilitators of patient care perceived by the caregivers of the elderly. The major themes that emerged as the facilitator for caregiving at home isolation were patient compliance with, and awareness about, the care required. Caregivers’ sense of responsibility was crucial in the home- and hospital-based treatment groups.

*“She has always taken care of us since childhood. I have to look after her….”* – said the sister (caregiver) of a 66-year-old widow getting treatment in home isolation. On the other hand, a 31-year-old female taking care of her father stated, *“He is my father! I am very comfortable taking care of him…But the problem is COVID-19 is not like any other normal diseases…,”* and got him admitted to a hospital.

Patients’ unwillingness to go to hospitals and non-compliance with the caregivers were important barriers as noted by caregivers of home-isolated patients. As per a 32-year-old female taking care of her mother in home-isolation treatment, “She (her mother) is very apprehensive of hospitals… So, unless something terrible happens, I can’t force her to go to a hospital.” In some instances, reports of deaths in hospitals shaped elderly patients’ non-compliance with hospital admission.

The caregivers who kept their patients at home felt hospital beds were scarce and lacked trust in health facilities. On the other hand, trust in government health facilities, fear of scarcity of hospital beds, and current availability were key drivers as per the caregivers of hospitalized elderly patients.

#### Loss, Risk, and Regret Aversion

The significant loss and regret aversion factors leading to treatment decisions are shown in Figure 1C. The respondents, perplexed by the uncertainty surrounding the disease outcome, were apprehensive of regretting any adverse effect from homecare. The uncertain nature of the disease also fueled the intense perception of loss. A 37-year-old daughter-in-law responded, “If we let him (father-in-law–the patient) stay at home, and then something happened, we could never have forgiven ourselves…” On the other hand, concern about being able to perform the last rites was one of the critical drivers for caregivers to keep elderly patients in home isolation. “If anything happens to him (father of the caregiver) in a hospital, the authority won't even let us have his dead body for the last rites,” – said a 30-year-old female caregiver. She believed, “…don't think God will forgive us if I don't perform the last rites for him (father of the caregiver).”

However, among the caregivers of the hospitalized patients, fear about the disease severity was a significant issue. The major factors behind hospitalization were avoiding the risk of adverse situations or any sudden emergency. Some caregivers felt that keeping a patient in home isolation when hospital beds are available would be risky considering the disease’s uncertain and deadly nature. “Thankfully, he got the much-needed hospital bed. I am still kind of dizzy when I remember how fast his condition worsened…” recalled the caregiver of a 63-year-old male who managed to avert the risk by getting admitted to a hospital. In most instances, the caregivers of the elderly who were hospitalized early exhibited lower risk tolerance. “If it were any other disease, I would not have admitted my mother… But with COVID-19, it is precarious… I do not feel good about taking this risk…” – confessed a 36-year-old female caregiver.

### Framework for Treatment Decisions

**FIGURE 2:**
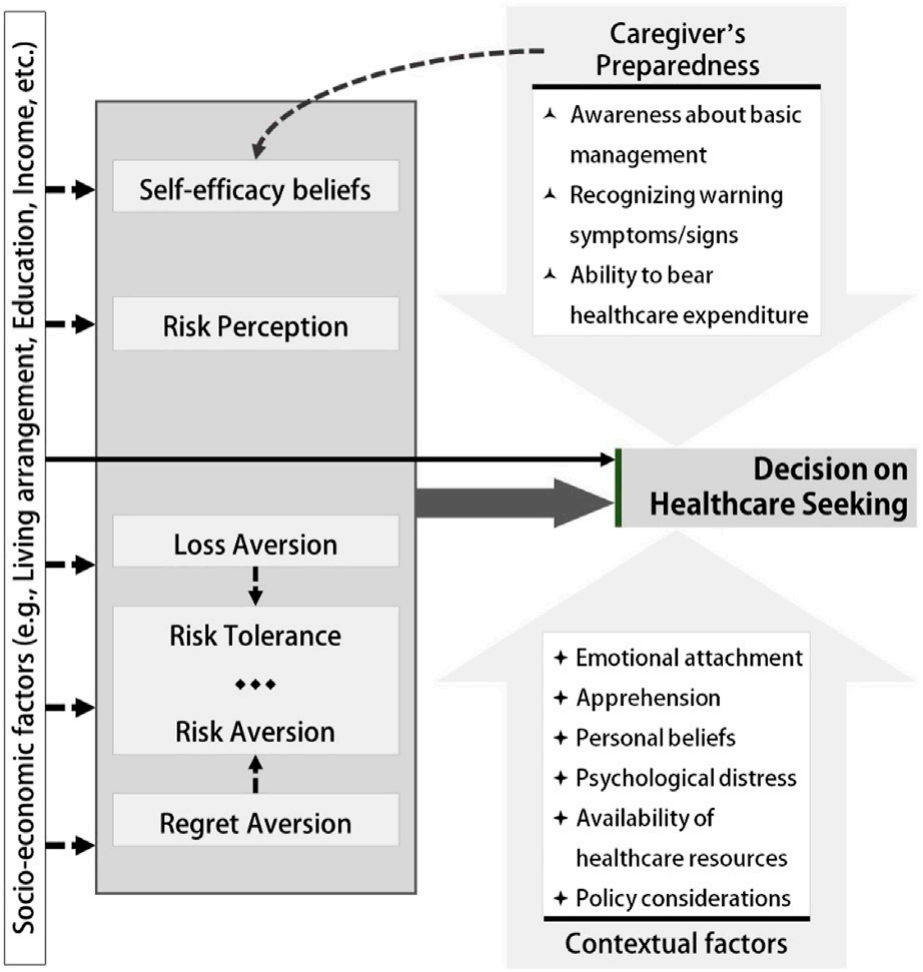
Model framework for caregiver’s decision-making regarding healthcare choice for the elderly patient (West Bengal, India. 2021). The broken arrows indicate probable effects. The solid arrows indicate relationships observed through quantitative or qualitative findings.

The model proposed in Figure 2 was devised after integrating the quantitative and qualitative findings. The outcome in this model is the decision related to healthcare seeking for the elderly. The model proposes four sets of predictors to predict this particular action under emergency/crisis situations. The primary predictors are the behavioral constructs that are often important in determining other health behaviors. Out of these behavioral constructs, the roles of self-efficacy, risk perception, and risk tolerance were tested in the quantitative part. In contrast, aversion phenomena, i.e., aversion to loss, risk, or regret, were established as essential constructs from the qualitative interviews. It was noted that risk aversion, explored qualitatively, essentially represented the other end of the risk tolerance spectrum of the caregivers. Qualitative findings suggest that loss and regret aversion often influence risk tolerance, apart from directly affecting the decision variable. The secondary predictors are different contextual factors and the factors depicting the caregiver’s preparedness. The contextual factors were mainly identified through qualitative explorations, whereas the quantitative and qualitative findings corroborated the caregivers’ preparedness issues. It was conceptualized that the caregiver’s preparedness somewhat influences the self-efficacy beliefs of a caregiver. The fourth set of predictors is the socio-economic factors of the caregiver, the elderly patient, and their family.

## Discussion

### Key Findings and the New Model Proposed

The role of different behavioral constructs in healthcare access-related decisions among the home-based primary caregivers of elderly COVID-19 patients was assessed through the adoption of the principles of case-control design at the community level. The majority of the caregivers of the elderly COVID-19 patients were aged <50 years and female. Compared to the hospitalized group, the home-isolated patients mostly had a lower *per capita* monthly income. Still, the caregivers of the home-isolated patients had a comparatively higher educational status. The clinical profile of the patients did not differ between the study groups. Caregivers were generally aware of the warning signs of COVID-19 illness and the requirements to keep an elderly patient in home isolation.

The model proposed in this study (Figure 2) specifically aimed at deciphering the factors influencing healthcare-seeking decisions by the caregivers of the elderly during emergencies/crises, such as during the COVID-19 pandemic. This model included certain new behavioral constructs, e.g., risk tolerance/risk aversion, regret aversion, loss aversion, self-efficacy, and risk perception. However, the socio-economic determinants have been considered to influence the predictor constructs and outcome decisions.

Self-efficacy beliefs of home-isolation-based care of the elderly were similar among the two groups of caregivers. The caregivers of the home-isolated elderly patients had a lower risk perception and a higher risk tolerance than the caregivers of the hospitalized patients. Disease severity, the stigma associated with keeping COVID-19 patients at home, and the cost of treatment were important issues along these constructs. However, in both the study groups the family members of the patients mostly influenced the decision of home isolation or hospitalization, though this effect was not observed in the multi-level model analysis.

The qualitative interviews documented that the caregiver’s sense of responsibility (manifestation of emotional bonding), awareness, and patient compliance with the caregiver were the primary facilitators for home-based treatment. However, the caregiver’s sense of responsibility was also a vital issue in the case of the decision of hospitalization. Caregivers of some of the home-isolated elderly patients lacked trust in health facilities. However, some caregivers considered the disease mild, and the optimism about uneventful recovery influenced their home isolation decision. Perception of the scarcity of hospital beds and the risk associated with the uncertain nature of the disease progression were key driving factors leading to early hospitalization of the patients. The perception of uncertainty was coupled with an aversion to the associated perception of loss and risk due to adverse outcomes. Regret about being unable to perform the last rites in the case of COVID-19-related death influenced the caregivers to decide against hospitalization.

### Findings in the Light of Relevant Literature

#### Role of Risk Perception

Golubeva et al. (2022), in their study in Russia, found that the most significant concerns reported by the family caregivers of the elderly were the health of the elderly, reduced access to healthcare services, and a fear of infecting the household with COVID-19 [27]. Similarly, the current study documented that higher risk perception about the elderly patient’s health status, infectivity, and scarcity of health resources lead the home-based family caregivers to decide against home isolation, independent of the clinical condition of the elderly [28]. The perceived threat of death of the elderly was a common decision influencer. In fact, preexisting co-morbidities were a factor behind caregivers’ perception of the higher risk [29].

There was a sense of increased anxiety among the caregivers when faced with a higher perception of risk in terms of severity, healthcare accessibility, and the treatment cost involved. The caregivers of COVID-19-infected patients in Hong Kong also demonstrated similar anxiety influenced by their risk perception of the disease [30]. In contrast to the current study findings, in a recent study (2019) regarding mental healthcare access among caregivers of adolescents with mental health conditions, it was noted that the financial barriers did not affect healthcare seeking [31]. However, caregivers’ consideration of the disease as mild and uneventful influenced their decision on home-isolation treatment. The home-based caregivers in the current study were mostly not concerned about getting infected, which was not the case elsewhere [30]. The respondent caregivers were concerned about carrying the infection to the household. In accordance, Anand et al. (2020), in their review, indicated that caregivers’ self-protection was a challenging issue [29]. The risk perception of the caregivers in terms of probable social discrimination contributed to the early hospitalization of elderly patients. This finding was consistent with the reports of stigma and discrimination in different parts of the country with the spread of the infection in the communities [32].

#### Role of Risk Tolerance

Home-based caregivers of elderly individuals increase their time for personalized care of the elderly, compensating for their tolerance to the uncertain nature of the COVID-19 illness [33]. Maffei et al. (2012) highlighted the role of tolerance and coping in decision-making during medical uncertainties [34]. The current study explored the role of risk tolerance in care seeking for COVID-19. The respondents recognized COVID-19 as an illness with an uncertain course and demonstrated tolerance to some scenarios while choosing home isolation for the elderly patient. While studying the risk tolerance of the informal caregivers for healthcare choices of patients with certain rare diseases, Morel et al. (2016) described tolerance to increasing disease severity and impairment or disability as factors behind treatment decisions [35]. Meisha et al. (2021) observed fear among the beneficiaries in seeking dental healthcare even for urgent needs during the COVID-19 pandemic, leading to higher pain tolerance [36]. In the current study, higher risk tolerance toward disease severity and social discrimination following home-based care were associated with home isolation of elderly patients. This was conceptually consistent as higher risk perception regarding them leads to early hospitalization. On the other hand, higher risk tolerance towards the chance of increased severity in the future was protective against the decision of home isolation. The probable explanation may be the time dependence in accepting the risk or a time-dependent shift in the acceptable threshold of benefit-risk trade-off. A similar temporal shift in acceptable threshold and risk tolerance was observed among patients receiving severe treatment adverse effects in exchange for progressive symptom relief [37].

#### Role of Perception About Regret and Response to Loss

A systematic review by Su et al. (2020) documented the influence of the fear of loss or regret among the home-based family caregiver in healthcare decision-making for the patient [38]. The caregiver’s perception of uncertainty was coupled with the aversion to the associated risk of adverse outcomes in the current study. To avoid the associated regret, caregivers often opted for early hospitalization following confirmation of COVID-19. As Bergmann and Wagner (2021) observed, to compensate for the uncertainty and perceived regret, informal caregivers of the elderly increased their time for personalized care, irrespective of their health status [33]. The constant fear of death of the elderly due to the COVID-19 illness while in home isolation was a key stressor and a significant source of perceived loss among the home-based family caregivers, similar to the findings of the qualitative exploration by Fajardo Ramos et al. (2021) [28]. The regret of not being able to perform the last rites in the case of COVID-19-related death influenced the caregivers to decide against hospitalization. This mainly was noted during the second wave, where there was a high death toll due to COVID-19 and media reports of difficulties in cremation. However, the sense of responsibility among the caregivers and the fear of regret associated with any adverse outcome from COVID-19 were the facilitators of early hospitalization of COVID-19 infected elderly patients.

#### Role of Self-Efficacy Beliefs

In their study on palliative care, Lamontagne et al. (2011) concluded that the healthcare choice decision largely depends on the self-efficacy of the arrangement of overall care provision [39]. In the current study, the authors have examined the different aspects of understanding comprehensive care and explored the self-efficacy items that significantly contribute to treatment choice. Treatment expenditure-related self-efficacy among the caregivers was associated with the home-isolation treatment of their elderly patients. This was consistent with the finding that those perceiving expenditure for home-based treatment as a risk were more prone to hospitalize their patients early. However, higher income was associated with not keeping a patient in home isolation. This may be explained by the difference in economic risk tolerance and health risk tolerance among the respondent families. The caregiver’s self-efficacy related to getting admission in the future as and when required was mostly against home-based treatment. This finding should be interpreted in light of the fact that despite higher risk tolerance toward progressive disease severity and uncertainty, caregivers decided against keeping their patients at home. The discourse may be explained by the higher risk perception about progressive disease severity, future bed scarcity, and the regret aversion phenomenon.

#### Caregiver’s Preparedness and Influences

Caregivers’ awareness about the regular measurement of oxygen saturation and patients reporting fever as their symptom was associated with home-isolation treatment. This was supported by the qualitative findings documenting the caregiver’s sense of responsibility and awareness as the significant facilitators for home-based therapy. The conclusion by Maffei et al. (2012) regarding the role of adequate information in enabling caregivers to make appropriate treatment decisions in medically uncertain situations was similar to the current findings [34]. Saah et al. (2021) noted in their study among the general population that during the COVID–19 pandemic, health-seeking behavior improved through increased health consciousness and regular health check-ups [40]. The patient’s compliance with the caregiver was another factor enabling the caregiver to decide among healthcare options, similar to the observation by Su et al. (2020) [38]. Su et al. (2020) also commented that family support was another key issue in home-based family caregivers’ decision-making process. However, in the case of COVID-19 illness, caregivers were often faced with the dilemma of the disease spreading among other family members. In the current study, elders from the joint family were comparatively more hospitalized than those from nuclear families. Although there is evidence of different normative influences on home-based family caregivers’ decision-making regarding the care of the elderly [41], in the multi-level model, the selected normative influencers did not have any statistically significant effect. The acuteness and uncertainty associated with the disease may have forced the caregivers to think more from a risk-driven perspective.

Caregivers of some home-isolated elderly patients lacked trust in the health facilities leading to home-based care irrespective of the clinical status. Prior hospital experience and mistrust of healthcare teams have already been documented as essential factors in healthcare seeking [42]. The CHARLS study findings from China demonstrated older patients’ inclination toward accessing care from top-tier institutions compared to community health institutions [43]. In the current study, some caregivers were convinced about better care provision in private hospitals. In contrast, others believed in the thoroughness of care provision of the public hospitals, which led to a decision in favor of hospital-based care for elderly patients.

### Strengths and Limitations

The current study is probably the first one in the Indian context to quantify different behavioral constructs and measure their effect on healthcare decision-making. The qualitative interviews helped to understand the contextual factors of the decision-making by the caregivers. The data collected during the pandemic posed a significant challenge due to the existing stigma, the threat of infection spread amidst imposed regulations, and the high level of anxiety at the community level. Despite the best efforts, survival bias might have been introduced during participant selection due to variable disease severity. Also, the extent of the survival bias may have been different during the two waves of COVID-19. However, assistance from the local non-governmental organizations and self-help groups helped access the study participants unbiasedly, as these organizations registered beneficiaries from all the sections of the communities during the pandemic, especially during the peak of the second wave. It must be noted that even during an emergency such as the COVID-19 pandemic, the welfare activities of the community organizations may have failed to reach some last-mile beneficiaries. Thus, a portion of the marginalized groups may have been under-represented. Still, the involvement of the local community-based organizations and their beneficiary enlistment strategies helped overcome the participant volunteering bias. This study analyzed the predictors from an individual caregiver’s perspective. The study did not clearly obtain the role of social capital and social support, although prior research has documented social capital as a crucial determinant in healthcare seeking [44]. Still, it can be argued that the distancing measures might have culminated in individually affected families being effectively cut off from their peers and the community.

### Conclusion

It is imperative to understand the behavioral factors that lead the beneficiaries and their caregivers to choose a particular healthcare option to improve healthcare access and promote equity in access through rational decision-making. In this study, the caregivers of the home-isolated elderly patients reported a lower income status and a higher educational level than the hospitalized group. Self-efficacy, risk perception, and risk tolerance related to different issues were critical factors behind the choice of healthcare decision. Apart from disease severity, and associated stigma, the cost of treatment was also an essential consideration along these behavioral constructs. When controlling for the socio-clinical status of the elderly patients and the behavioral factors of the home-based caregivers, the normative influencers did not have any significant role in influencing the treatment decision. Thus, it may be inferred that in uncertain and unprecedented situations, the primary decision-maker’s perception of the disease, tolerance towards potential untoward incidences, and self-confidence are the most significant factors for choosing and accessing a healthcare option. The qualitative evidence corroborated these findings. The loss, risk, and regret aversion phenomenon and individual perception about the efficiency of the health system at the intersection of variable severity perception motivated the caregivers to actively act in choosing a particular treatment option. The evidence is not limited to the COVID-19 pandemic context only. It will also help prepare for the next health emergency or any pandemic with a beneficiary behavior-oriented approach, as it will help in understanding the optimum institutional capacity requirements. The findings presented in this study can help prepare for healthcare access equity in any emergency or outbreak situation. Interventions should be targeted towards risk tolerance and perception in an integrated way based on the homogeneity of the care providers’ awareness levels and risk and regret aversion among the target population to achieve appropriateness and equity in healthcare seeking during emergencies. The behavioral constructs presented in this model can be further tested for their relative contribution to the choice of treatment. At the policy level, cost-effective behavioral interventions based on the described model can be designed to optimize the equilibrium of healthcare needs and delivery.
