# Supplementary material for: Home-Isolation Care in Newly COVID-19-Positive Elderly Patients: A Caregiver-Centric Explanatory Framework
Source: Int J Public Health. 2023 Jul 19;68:1606060. doi: 10.3389/ijph.2023.1606060 (PMC10394230; doi:10.3389/ijph.2023.1606060)
Supplement: Supplementary file 1 [file Table1.docx]

**Table S1. Socio-demographic information of the participants. (West Bengal, India. 2021)**

|  | Home isolation patients (n = 1412) | Hospital admission patients (n = 1392) | P-value ^a^ |
| --- | --- | --- | --- |
| Age of the caregiver in years |  |  | 0.635 |
| < 30 | 57 (4.04) | 54 (3.88) |  |
| 30 – 39 | 859 (60.84) | 826 (59.34) |  |
| 40 – 49 | 484 (34.28) | 502 (36.06) |  |
| 50 – 59 | 9 (0.64) | 5 (0.36) |  |
| ≥ 60 | 3 (0.21) | 5 (0.36) |  |
| Gender of the caregiver |  |  | 0.984 |
| Male | 321 (22.73) | 316 (22.70) |  |
| Female | 1091 (77.27) | 1076 (77.30) |  |
| Religion |  |  | 0.630 |
| Hinduism | 1176 (83.29) | 1172 (84.20) |  |
| Islam | 206 (14.59) | 187 (13.43) |  |
| Others ^b^ | 30 (2.12) | 33 (2.37) |  |
| Type of family |  |  | 0.930 |
| Nuclear | 755 (53.47) | 742 (53.30) |  |
| Joint | 657 (46.53) | 650 (46.70) |  |
| Per capita monthly family income (in INR) ^c^ |  |  | 0.000 |
| ≤ 5000.00 | 564 (39.94) | 150 (10.78) |  |
| 5000.01 – 8000.00 | 339 (24.01) | 360 (25.86) |  |
| 8000.01 – 13750.00 | 126 (8.92) | 623 (44.76) |  |
| >13750.00 | 383 (27.12) | 259 (18.61) |  |
| Education of the caregiver |  |  | 0.025 |
| Completed secondary or below | 421 (29.82) | 463 (33.26) |  |
| Completed Higher-secondary | 525 (37.18) | 532 (38.22) |  |
| Graduate or above | 466 (33.00) | 397 (28.52) |  |
| Currently an earning member |  |  |  |
| The patient | 112 (7.93) | 90 (6.47) | 0.133 |
| The caregiver | 547 (38.47) | 539 (38.72) | 0.992 |

‘n’ represents the number of participants in each study group. The figures within parentheses represent the column percentage of each cell. INR: Indian Nationalized Rupees

^a^ P-values calculated by chi-squared test, ^b^ other religions include Christianity and Jainism, ^c^ Per-capita monthly income of the patient’s family has been divided into quartiles.
