# Supplementary material for: Home-Isolation Care in Newly COVID-19-Positive Elderly Patients: A Caregiver-Centric Explanatory Framework
Source: Int J Public Health. 2023 Jul 19;68:1606060. doi: 10.3389/ijph.2023.1606060 (PMC10394230; doi:10.3389/ijph.2023.1606060)
Supplement: Supplementary file 2 [file Image2.pdf]

Home-Isolation Care in Newly COVID19-positive Elderly Patients: A Caregiver-Centric Explanatory Framework

Supplementary Information

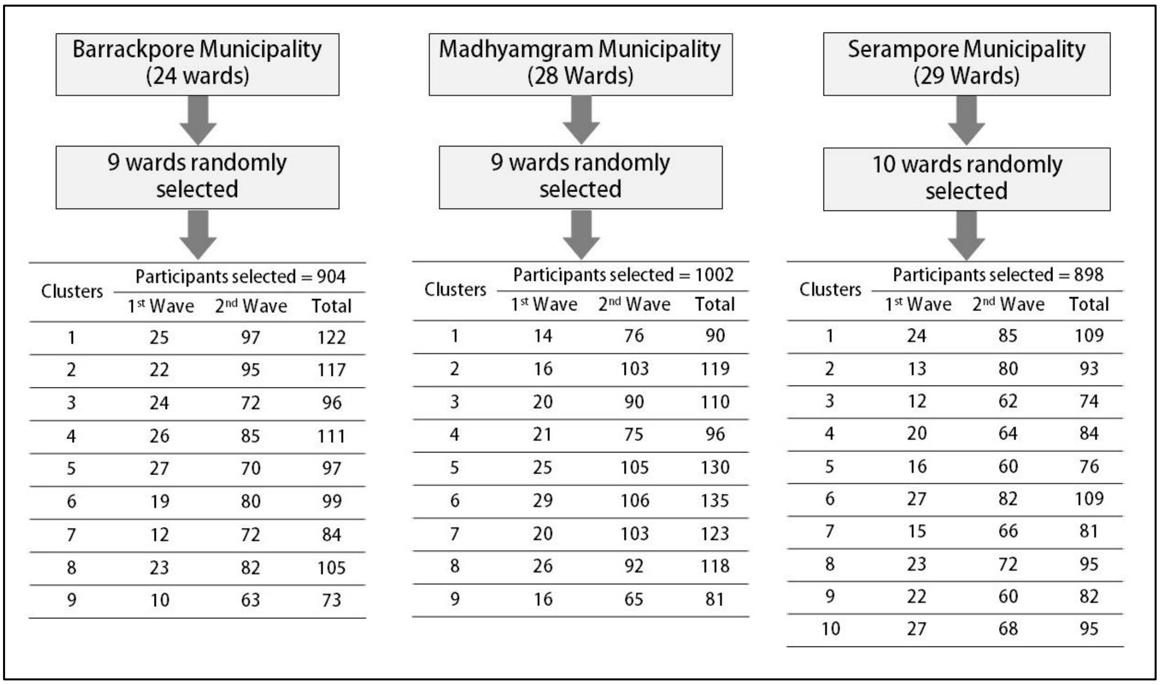

Clusters	Participants selected = 904		
	1st Wave	2nd Wave	Total
1	25	97	122
2	22	95	117
3	24	72	96
4	26	85	111
5	27	70	97
6	19	80	99
7	12	72	84
8	23	82	105
9	10	63	73

Clusters	Participants selected = 1002		
	1st Wave	2nd Wave	Total
1	14	76	90
2	16	103	119
3	20	90	110
4	21	75	96
5	25	105	130
6	29	106	135
7	20	103	123
8	26	92	118
9	16	65	81

Clusters	Participants selected = 898		
	1st Wave	2nd Wave	Total
1	24	85	109
2	13	80	93
3	12	62	74
4	20	64	84
5	16	60	76
6	27	82	109
7	15	66	81
8	23	72	95
9	22	60	82
10	27	68	95

Figure S2. Participant selection strategy in the three selected study areas. (West Bengal, India. 2021)
