# Supplementary material for: Home-Isolation Care in Newly COVID-19-Positive Elderly Patients: A Caregiver-Centric Explanatory Framework
Source: Int J Public Health. 2023 Jul 19;68:1606060. doi: 10.3389/ijph.2023.1606060 (PMC10394230; doi:10.3389/ijph.2023.1606060)
Supplement: Supplementary file 3 [file Table2.docx]

## **Table S2. Basic demographic information about the participants included in the qualitative part. (West Bengal, India. 2021)**

|  | Home isolation patients (n = 20) | Hospital admission patients (n = 15) |
| --- | --- | --- |
| Gender of the patient under care of the respondent |  |  |
| Male | 9 | 8 |
| Female | 11 | 7 |
| Gender of the caregiver (respondent) |  |  |
| Male | 7 | 5 |
| Female | 13 | 10 |
| Age of the patient (in years) |  |  |
| 60 - 69 | 12 | 9 |
| 70 - 79 | 7 | 4 |
| ≥ 80 | 1 | 2 |
| Age of the caregiver (in years) |  |  |
| < 30 | 4 | 1 |
| 30 – 39 | 8 | 9 |
| 40 – 49 | 7 | 3 |
| 50 – 59 | 0 | 2 |
| ≥ 60 | 1 | 0 |
| Relationship of the caregiver with the patient |  |  |
| Father | 7 | 4 |
| Father-in-law | 2 | 3 |
| Mother | 4 | 6 |
| Mother-in-law | 6 | 1 |
| Husband | 0 | 1 |
| Sister | 1 | 0 |

‘n’ represents the number of participants in each study group included in the qualitative interviews.
