# Supplementary material for: Home-Isolation Care in Newly COVID-19-Positive Elderly Patients: A Caregiver-Centric Explanatory Framework
Source: Int J Public Health. 2023 Jul 19;68:1606060. doi: 10.3389/ijph.2023.1606060 (PMC10394230; doi:10.3389/ijph.2023.1606060)
Supplement: Supplementary file 4 [file Table3.docx]

**Table S3. Clinical profile of the patients. (West Bengal, India. 2021)**

|  | Home isolation patients (n = 1412) | Hospital admission patients (n = 1392) | P-value ^a^ |
| --- | --- | --- | --- |
| Current symptoms ^b^ |  |  |  |
| Loss of taste | 489 (34.63) | 470 (33.76) | 0.628 |
| Loss of smell | 603 (42.71) | 590 (42.39) | 0.864 |
| Fever | 913 (64.66) | 856 (61.49) | 0.082 |
| Body-ache | 1056 (74.79) | 1041 (74.78) | 0.999 |
| Shortness of Breath | 245 (17.35) | 257 (18.46) | 0.443 |
| Diarrhea | 360 (25.50) | 370 (26.58) | 0.513 |
| Diagnosed co-morbidities ^b^ |  |  |  |
| Hypertension | 592 (41.93) | 579 (41.59) | 0.859 |
| Diabetes | 549 (38.88) | 538 (38.65) | 0.900 |
| COPD/ Asthma | 528 (37.39) | 484 (34.77) | 0.148 |
| Thyroid disorder | 408 (28.90) | 387 (27.80) | 0.521 |
| Cancer | 239 (16.93) | 235 (16.88) | 0.975 |
| Mental illness | 276 (19.55) | 237 (17.03) | 0.084 |

‘n’ represents the number of participants in each study group. The figures within parentheses represent the column percentage of each cell.

^a^ P-values calculated by chi-squared test, ^b^ multiple responses
