# Supplementary material for: Home-Isolation Care in Newly COVID-19-Positive Elderly Patients: A Caregiver-Centric Explanatory Framework
Source: Int J Public Health. 2023 Jul 19;68:1606060. doi: 10.3389/ijph.2023.1606060 (PMC10394230; doi:10.3389/ijph.2023.1606060)
Supplement: Supplementary file 5 [file Image1.pdf]

Home-Isolation Care in Newly COVID19-positive Elderly Patients: A Caregiver-Centric Explanatory Framework

Supplementary Information

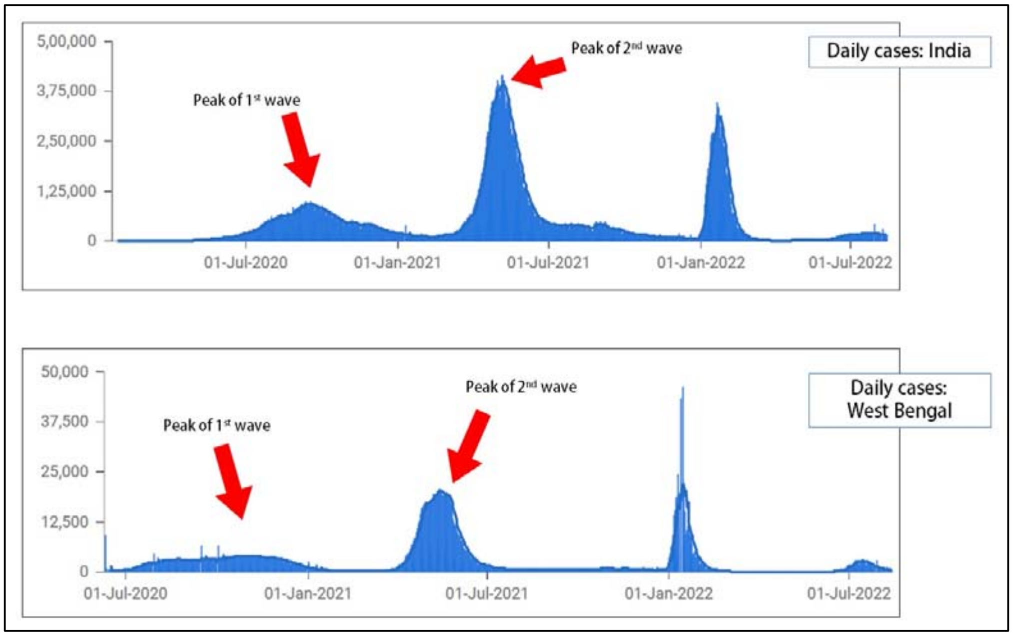

Figure S1. Burden of daily new COVID-19 cases in India and West Bengal. (Data Source: JHU CSSE COVD-19 Data[1])

References:

1. Dong E, Du H, Gardner L. An interactive web-based dashboard to track COVID-19 in real time. The Lancet Infectious Diseases 2020;20(5):533–4.
